# Integration of surgical and Ayurvedic interventions in the treatment of Fournier's Gangrene: A Case Report

**DOI:** 10.1016/j.jaim.2026.101416

**Published:** 2026-10-12

**Authors:** Dixit Donga, Hemant Toshikhane, Vikram Singh, Aswin Haridas

**Affiliations:** Department of Shalya Tantra, Parul Institute of Ayurved, Parul University Vadodara, Gujrat, India

**Keywords:** Fournier's gangrene, *Kotharoga*, Surgical debridement, Split-thickness skin grafting, Case report

## Abstract

Fournier's gangrene is a rare, life-threatening form of necrotizing fasciitis affecting the perineum and genital region. In Ayurveda, it correlates with *Kotharoga*, as both share symptoms such as severe tissue necrosis, pus discharge, and a foul odor. This case report discusses a 50-year-old male who presented with fever, severe scrotal pain, blackish discoloration, and purulent discharge two days after undergoing incision and drainage (I&D) of a perianal abscess. The case was diagnosed based on clinical examination and imaging studies. Broad-spectrum antibiotics, along with herbo-mineral medicines and *Rasa Aushadhi*, were administered immediately. Urgent and extensive surgical debridement was performed to remove necrotic and infected tissue. Advanced wound management included collagen granule application and split-thickness skin grafting. The signs of systemic toxicity subsided within 48 hours. By day 7, purulence and odor had markedly decreased, and healthy granulation covered more than 70% of the wound bed. Collagen application accelerated healthy granulation, and skin graft take exceeded 90% within three weeks, restoring scrotal integrity without complications. Early recognition, aggressive surgical intervention, and multidisciplinary care are crucial for improving prognosis. This case highlights the importance of innovative wound care techniques in optimizing integrative treatment strategies for Fournier's gangrene.

## 1 Introduction

Fournier's gangrene (FG) is a rapidly progressive, life-threatening necrotizing fasciitis of the perineal, genital, and perianal regions characterized by fulminant soft-tissue infection, thrombosis of subcutaneous vessels, and widespread fascial necrosis [1]. Named after Jean-Alfred Fournier, the condition is typically polymicrobial, with synergistic aerobic and anaerobic organisms (*streptococci, staphylococci, Bacteroides,* and *Clostridium species*) that produce toxins and enzymes, resulting in rapid tissue destruction and gas formation. The resulting pathological cascade involves microvascular occlusion, ischemia, and liquefactive necrosis, with systemic inflammatory response and sepsis common in advanced disease [2].

The most prominent risk factors include diabetes mellitus, chronic alcoholism, immunosuppression (such as corticosteroid use and HIV infection), obesity, chronic kidney disease, malignancy, and peripheral vascular disease [3]. Typical portals of entry are perianal infections (abscess, fistula), urogenital sources (urethral strictures, catheterization, periurethral infections), traumatic skin breaches, and postoperative or iatrogenic injuries. Early clinical signs may be subtle (pain out of proportion, edema, erythema, and crepitus), underscoring the need for a high index of clinical suspicion and prompt intervention. Fournier's gangrene is uncommon but carries substantial morbidity and mortality. The incidence is higher in men and increases with age, peaking during the sixth and seventh decades of life. A strong male predominance has been consistently reported, although female cases occur and may present atypically. Diabetes is present in approximately half of cases and worsens outcomes. Despite advances in critical care and surgery, reported mortality remains high often ranging from 20 to 40%, driven by delays in debridement, the extent of necrosis, septic shock, and comorbidity burden [4]. Standard management entails immediate administration of broad-spectrum antibiotics, aggressive hemodynamic resuscitation, urgent and repeated surgical debridement, timely reconstruction with hyperbaric oxygen therapy considered on a case-by-case basis [5].

In Ayurveda, Fournier's gangrene can be correlated with *Kotharoga*, which is characterized by blackish discoloration, putrefaction, pus formation, and foul odor. The *Samprapti* (pathogenesis) of *Kotha* arises from *Margavarana* (obstruction of bodily channels) and *Dhatu Kshaya* (tissue depletion). The term *Marga* refers to the channels that transport nutrients throughout the body, whereas *Avarana* denotes obstruction of these channels. In this condition, vitiated *Kapha* and *Pitta dosha* accumulate in the channels and obstruct the natural movement (*gati*) of *Vata dosha*. This interference disturbs circulation and nourishment, ultimately leading to tissue necrosis and gangrenous changes. The *Samprapti-ghataka* (components of pathogenesis) involve vitiated *Kapha* and *Pitta* with predominance of *Kleda* (excessive discharge), along with *Srotodusti* (vitiation of channels) in the form of *Sanga* (obstruction) [6]. For its management, *Acharya Sushruta* advocated *Chedana* (excision), *Vrana Sodhana* (wound cleansing), and *Ropana* (healing).

This case is clinically and academically significant as it demonstrates the integration of modern surgical care with Ayurvedic therapy in managing Fournier's gangrene, ensuring both safety and efficacy in a high-mortality condition. Diagnosis was confirmed through clinical signs (pain, edema, necrosis, purulent discharge), ultrasonography (diffuse scrotal wall edema), and microbiology (*Escherichia coli* growth). The absence of comorbidities allowed a clear assessment of integrative outcomes. This report enriches existing literature by showing that timely surgical debridement combined with selected Ayurvedic formulations can accelerate wound healing, minimize systemic toxicity, and promote complete functional recovery in Fournier's gangrene.

## 2 Clinical history

A 50-year-old non-smoker and non-alcoholic male patient presented with a history of incision and drainage (I&D) of a perianal abscess performed two days earlier at a private medical college hospital. Within 24 hours after the procedure, he developed worsening perianal and scrotal pain, followed by the onset of high-grade fever (102 °F). Within the next 24 hours, he developed progressive scrotal edema with dusky blackish discoloration of the skin, and penile swelling developed. Copious purulent drainage persisted from the perianal wound site during this period. The patient denied any history of hypertension, diabetes, or other significant medical, surgical, or familial illness.

## 3 Clinical examination

On inspection, an approximately 10 cm perianal wound extending from the base of the scrotum to the right perianal region, with purulent discharge, slough, blackish discoloration, localized warmth, tenderness, and penile shaft swelling Fig. 1a.

**Fig. 1:**
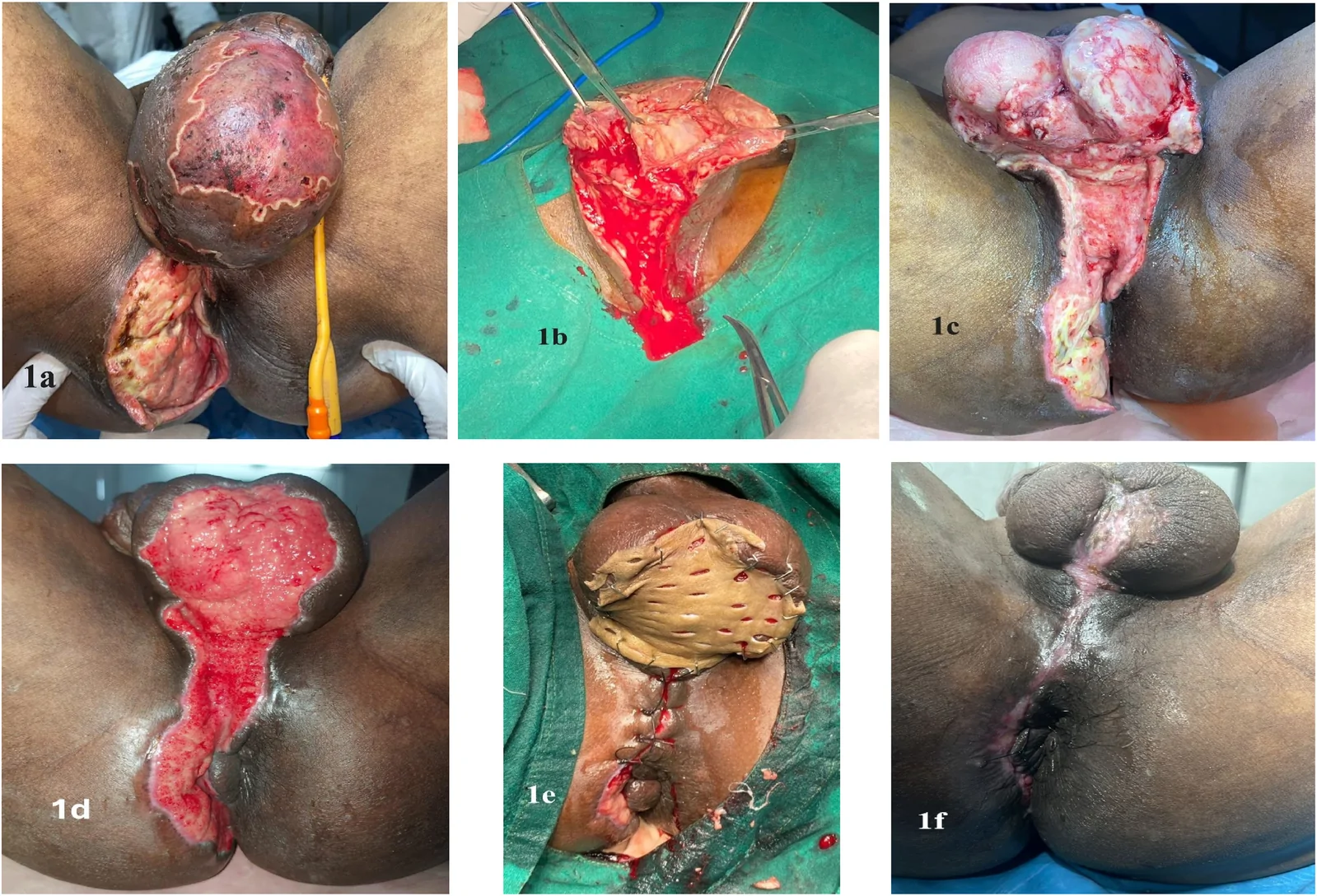
Therapeutic Interventions: (**a**) Wound on 1st Day, (**b**) Wound during Debridement, (**c**) Wound on 8th Day, (**d**) Wound on 28th Day, (**e**) STSG procedure + Perianal wound closure, (**f**) Wound at first follow-up, wound healed with contracture.

The patient's vital signs on admission were temperature 102 °F, pulse rate 100/min, and blood pressure 130/70 mmHg. Systemic examination findings were within normal limits at the time of admission.

## 4 Timeline

The patient had been asymptomatic until 10 days earlier, when he noticed a perianal swelling. He applied a *Madanphala* (*Randia dumetorum Lam*) paste locally, following which he experienced worsening pain and purulent discharge the next day. He subsequently underwent incision and drainage of the perianal abscess at an outside facility. Post procedure, he developed high-grade fever and severe pain in the perianal and scrotal regions. Within 24 hours, progressive scrotal edema with dusky blackish discoloration and associated penile swelling developed, accompanied by persistent purulent discharge. Consequently, the patient presented to our hospital for further evaluation and management.

## 5 Diagnosis

Fournier's gangrene (FG) is primarily diagnosed through clinical evaluation, supported by laboratory investigations and imaging studies [7]. The differential diagnoses included necrotizing fasciitis of the deep fascial planes, gas gangrene involving the more superficial soft tissues, and a localized scrotal abscess.

Ultrasonography revealed diffuse edema and thickening of the scrotal wall and septum with minimally increased vascularity, suggestive of infective or passive edema.

Magnetic resonance imaging (MRI) demonstrated post-operative changes in the right perianal and right intersphincteric region, with diffuse scrotal edema but no obvious fluid collection. Diffuse edema was also noted in the bilateral spermatic cords, raising the possibility of funiculitis, along with minimal fluid in the scrotal sac.

Relevant diagnostic investigations are summarized in Table 3.

## 6 Therapeutic intervention

A detailed day-wise account of surgical interventions, antimicrobial therapy, wound care, and Ayurvedic management is presented in Table 1.

**Table 1:** Therapeutic intervention timelines.

Therapeutic Intervention Timelines		
Drug/Route/Dose	Day	Procedure
Inj. Piperacillin + Tazobactam 4.5 g IV TDS (as per the antimicrobial susceptibility report)	Day 1 to Day 7	Daily dressing with povidone-iodine.
Surgical debridement (Procedure 1)	Day 8	-
Inj. Metronidazole 500 mg IV TDS	Day 8 to Day 21	Daily dressing with povidone-iodine solution, collagen particles application, chlorhexidine gauze [8].
Split-thickness skin grafting (STSG) + Peri anal wound closure (Procedure 2)	Day 30	-
Tab Cefixime + Clavulanic acid 325 mg, 1 tablet twice daily after food	Day 30 to Day 35	The STSG wound was cleaned with normal saline and dressed with human placenta extract gel and chlorhexidine gauze.
Perianal suture removal	Day 40	
Dressing	Until day 60	
Patient discharged	Day 61	-
Follow-up	Day 67	Complete healing achieved.

*Rasamanikya* 50 mg + *Gandhak Rasayana* 500 mg + *Amalaki Rasayana* 500 mg + *Praval Panchamrit Rasa* 500 mg, administered orally in two divided doses daily after food with water.		Day 1 to Day 15
Tab. *Arogyavardhani Vati,* 2 tablets twice daily after food, and Tab. *Nimbadi Guggulu,* 2 tablets twice daily after food with warm water.		Day 16 to Day 67

### 6.1 Procedure 1: surgical debridement

The procedure was performed under spinal anesthesia after fitness clearance, with informed consent obtained.

#### 6.1.1 Indication

Excision of necrotic scrotal tissue to control infection and prevent disease progression [9].

#### 6.1.2 Operative procedure

A midline scrotal incision exposed necrotic tissue, which was excised down to the tunica vaginalis, sparing healthy testes and spermatic cords. Hemostasis was achieved, and the wound was irrigated. Primary closure was not possible due to tissue loss; the wound was packed with sterile gauze (Fig. 1b). Postoperatively, the patient remained stable and was managed per protocol.

### 6.2 Procedure 2: STSG procedure + perianal wound closure

#### 6.2.1 Indications

STSG was performed to reconstruct the scrotal defect, and the perianal wound was closed primarily.

#### 6.2.2 Findings

On examination, a 5 × 6 cm scrotal defect with exposed underlying structures and a perianal wound with granulation tissue up to the margins were noted, requiring surgical closure.

#### 6.2.3 Operative procedure

A 7 × 7 cm split-thickness skin graft was harvested from the right lateral thigh using a dermatome, perforated to prevent seroma formation, and kept in normal saline. The donor site was dressed appropriately. The scrotal defect was then irrigated, debrided, and the edges undermined to facilitate graft placement. A 5 × 6 cm graft was applied over the scrotum, extending from the ventral aspect of the penis to the anterior defect, and secured with skin staples. The perianal wound was closed in layers with non-absorbable sutures, followed by sterile dressings, and no complications occurred.

## 7 Outcome

### 7.1 Intervention adherence and tolerability

Treatment adherence was assessed through clinical monitoring, laboratory investigations, symptom tracking, and medication compliance, with no major complications observed. Following debridement, symptoms such as pain, pus discharge, and swelling resolved completely, and the wound demonstrated rapid healing following application of collagen and daily dressings. Wound healing was documented through serial clinical examinations and photographs, ensuring effective treatment adherence. After split-thickness skin grafting (STSG), complete wound closure was achieved without complications. Laboratory investigations confirmed the resolution of infection, with WBC counts normalizing within 10 days. Regular monitoring confirmed the safety of the herbo-mineral formulations when used in proper dosage and under supervision. Microbial follow-up cultures were not performed; however, clinical signs of infection were closely monitored and resolved completely, and laboratory parameters (WBC counts) normalized during the treatment period.

Laboratory investigations are summarized in Table 2.

**Table 2:** Laboratory investigations.

Blood Reports	25/05/23 (Admission)	27/05/23	30/05/23	02/06/23	12/06/23
Hemoglobin (g/dL)	13.2	11.5	10.8	10.9	10.7
WBC (mm^3^)	31,700	22,400	17,100	10,900	7500
Platelet (mm^3^)	225,000	121,000	159,000	278,000	243,000
CRP (mg/L)	232.60	-	-	-	-
Tissue Culture and Sensitivity	*Escherichia coli*	-	-	-	-

- = Not Available.

The serial assessment Table 3 of scrotal and perianal wound parameters is presented in Table 3.

**Table 3:** Assessment of scrotal and perianal wound parameters.

Wound parameters	Day 0 (Fig. 1a) Fournier Gangrene	Day 8 (Fig. 1c) After debridement	Day 28 (Fig. 1d) After collagen dressing	Day 30 (Fig. 1e) STSG & perianal closure	Day 67 (Fig. 1f) Follow up
Scrotal wound					
Scrotal skin integrity	Extensive necrosis, loss of tissue	Debrided with exposed base	Granulating wound bed, partial epithelialization	Grafted zone forming	Healed with full coverage
Texture	Dry, necrotic	Moist raw surface	Soft, granulating	Firm, grafted	Smooth and supple
Color	Blackish and dusky	Red and raw	Healthy red-pink granulation	Pink, well-integrated graft	Pinkish near-normal tone
Swelling	Severe edema	Moderate edema	Mild swelling	Minimal swelling	Resolved
Pain/sensation	Severe pain	Moderate pain on manipulation	Mild discomfort	Very mild	No pain, normal sensation
Discharge	Purulent and foul-smelling	Serous with slough	Minimal serous	None visible	None
Odor	Foul *(Visra Gandha)*	Mild odor	None	None	None
Perianal Wound					
Size	20 × 20 cm (400 cm^2^)	18 × 15 cm (270 cm^2^)	10 × 10 cm (100 cm^2^)	6 × 5 cm (30 cm^2^)	Healed (0 cm^2^)
Reduction time	-	16.25cm^2^/day	8.5cm^2^/day	35cm^2^/day	0.8cm^2^/day
Shape	Irregular, extensive	Irregular oval	Localized oval	Narrow linear	Linear, healed
Depth	2 cm	1.5 cm	0.5cm	0.2 cm	-
Discharge	Purulent, foul-smelling	Serous with slough	Minimal	None	None
Edge	Undermined, necrotic	Sloping	Approximating	Defined	Healed, well-defined
Floor	Necrotic, sloughy tissue	*PGT	*RGT	*RGT	Epithelialized
Odor	Foul odor	Faint odor	No odor	None	None
*BWAT score [10]	50	38	28	16	13
VAS score	4	3	2	1	0

• *BWAT: Bates-Jensen Wound Assessment Tool.

• *VAS: Visual Analogue Scale.

• *PGT: Purulent Granulation Tissue.

• *RGT: Red Healthy Granulation Tissue.

No adverse events were observed.

### 7.2 Follow-up

By day 67, the patient showed no signs of infection or discharge and had achieved complete wound healing and recovery. The patient regained normal mobility and urination, with a marked improvement in overall quality of life. Fig. 1f.

## 8 Discussion

This case highlights the importance of prompt and comprehensive management of Fournier's gangrene. A 50-year-old male developed scrotal necrosis following incision and drainage of a perianal abscess, emphasizing the need for early recognition and timely intervention. Management was based on the Ayurvedic principle of *Chedana* (excision), one of the eight major surgical procedures described by *Sushruta*, and indicated in conditions such as *Kotha*, *Bhagandara*, and *Granthi* [11]. Surgical debridement corresponded to the first principle of *Shodhana*, facilitating complete removal of necrotic tissue and control of local infection. Local wound cleansing was achieved through the use of medicated dressings, such as povidone-iodine [12] and collagen granules [13]. In addition, Ayurvedic preparations like *Rasamanikya* [14,15] and *Gandhak Rasayana* [15,16] were administered, which possess antimicrobial (*Krimighna)* and detoxifying properties, thereby supporting the wound-cleansing (*Shodhana*) process.

The second principle, *Ropana* (healing), was achieved through the local application of collagen granules, which promoted the formation of granulation tissue and epithelialization Oral formulations, including *Amalaki Rasayana* [17] and *Praval Panchamrit Rasa*, were administered to pacify *Pitta* and *Rakta dosha*, thereby reducing inflammation and promoting tissue healing. The third principle, *Rasayana*, focused on systemic rejuvenation and immune enhancement. *Gandhak Rasayana* and *Amalaki Rasayana* acted as immunomodulators, improved tissue perfusion, prevented recurrent infection, and supported *dhatu poṣhana* (tissue nourishment), facilitating recovery from extensive tissue loss. In addition, *Nimbadi Guggulu* [18] and *Arogyavardhani Vati* provided antimicrobial (*Krimighna*) and blood-purifying (*Raktashodhana*) effects, reducing pus formation, foul odor (*Visra Gandha*), and microbial proliferation. Together, these interventions complemented surgical management, enhanced wound healing, minimized the risk of recurrence and, along with STSG and placental extract gel [19], restored scrotal integrity with satisfactory graft uptake.

Clinically, systemic toxicity resolved within 48 hours of treatment. By day 7, purulent discharge and foul odor had markedly decreased, and healthy granulation tissue covered more than 70% of the wound bed. STSG, performed on day 30, resulted in more than 90% graft uptake within three weeks, resulting in successful restoration of scrotal integrity without complications. Hematological parameters, including WBC count, platelet count, and CRP levels, normalized in parallel with clinical recovery, reflecting effective infection control. Overall, these findings highlight the synergistic effect of timely debridement, appropriate medication, structured wound care, and staged reconstruction in achieving successful recovery in Fournier's gangrene.

This case supports the role of integrative Ayurvedic and surgical management in Fournier's gangrene by demonstrating favorable wound healing, effective infection control, and systemic recovery. Early diagnosis, prompt debridement, structured wound care, and staged reconstruction, together with Ayurvedic therapy, contributed to stable vital signs, absence of systemic sepsis, and successful clinical recovery. However, delayed presentation, underlying comorbidities, and increasing antibiotic resistance remain important challenges. Prevention of such complications begins during the initial I&D procedure. The abscess should be incised at its most dependent point using a Hilton or cruciate approach to establish an adequately large opening, thoroughly curette all necrotic debris, and place a suitable drain. Meticulous technique and diligent postoperative wound care preserve patency, prevent residual sepsis and fistula formation, and markedly reduce the risk of progression to life-threatening Fournier's gangrene.

## 9 Conclusion

This case contributes to the emerging literature on integrative management of life-threatening infections. The favorable outcome supports the hypothesis that Ayurvedic interventions when judiciously combined with surgical intervention and advanced wound care may enhance recovery and reduce complications in Fournier's gangrene. This report, though limited to a single patient, provides a novel foundation for future controlled clinical studies.

## 10 Patient's perspective

“When I was admitted, I had severe scrotal pain, fever, blackish discoloration, swelling, purulent discharge, and difficulty walking. After treatment, my pain subsided, swelling reduced, the wound healed completely, and I regained strength with no signs of infection or discomfort. The patient subsequently to normal daily activities.”

## Informed consent

Written permission for the publication of this case study was obtained from the patient.

## Declaration of patient consent

The authors certify that they have obtained all appropriate patient consent forms. In this form, the patients have given their consent for their images and other clinical information to be reported in the journal. The patients understand that their names and initials will not be published, and due efforts will be made to conceal their identity.

## Data availability statement

All collected data, including recordings of measurements, are properly stored physically or electronically as appropriate. They are stored without personal identifiers with the corresponding author, who can make them available under appropriate circumstances.

## Author contributions

Dr. Dixit Donga: Conceptualization, Methodology/Study design, Formal analysis, Investigation, Resources, Data curation, Writing – original draft, Writing – review and editing, Visualization, Supervision, Project administration, Funding acquisition.

Dr. Hemant Toshikhane: Conceptualization, Methodology/Study design, Formal analysis, Investigation, Writing – review and editing, Visualization, Supervision, Project administration, Funding acquisition.

Dr. Vikram Singh: Conceptualization, Methodology/Study design, Formal analysis, Investigation, Writing – review and editing, Visualization, Supervision, Project administration, Funding acquisition.

Dr. Aswin Haridas: Conceptualization, Methodology/Study design, Formal analysis, Investigation, Writing – review and editing, Visualization, Supervision, Project administration, Funding acquisition.

## Declaration of generative AI in scientific writing

The authors utilized ChatGPT for language editing assistance. After the use of AI, they thoroughly reviewed and refined the content, taking full responsibility for the final publication.

## Funding sources

None

## Declaration of competing interest

The authors declare that they have no known competing financial interests or personal relationships that could have appeared to influence the work reported in this paper.
